# Application of a nerve stereoscopic reconstruction technique based on ultrasonic images in the diagnosis of neuralgic amyotrophy

**DOI:** 10.3389/fphys.2023.1201275

**Published:** 2023-09-18

**Authors:** Miao Yu, Wenquan Ding, Guoqing Shao, Miaozhong Li, Xiaoling Zhou, Linhai Liu, Xueyuan Li

**Affiliations:** ^1^ Department of Hand Surgery, Ningbo No. 6 Hospital, Ningbo, China; ^2^ Department of Plastic Reconstructive Surgery, Ningbo No. 6 Hospital, Ningbo, China; ^3^ Department of Ultrasonic Medicine, Ningbo No. 6 Hospital, Ningbo, China

**Keywords:** constriction, nerve torsion, neuralgic amyotrophy, neuropathic, application

## Abstract

**Objective:** To propose a nerve stereoscopic reconstruction technique based on ultrasound imaging for site diagnosis, intuitive reflection of disease severity, and classification of neuralgic amyotrophy (NA).

**Methods:** We enrolled 44 patients with NA who underwent high-frequency ultrasonography examination. Multiple sites on the normal side and the affected side were scanned to calculate the ratio of the cross-section area (CSA) of the affected side to the normal side at each location measured, i.e., the cross-section area swelling ratio (CSASR). The CSASR of 44 patients and 30 normal controls was analyzed to determine their threshold value for the diagnosis of NA. Then, ultrasound images of the cross-section were used to reconstruct the stereoscopic model of the nerve on the affected side and the normal side. Using the CSASR values in each measurement location, a CSASR stereoscopic model was developed.

**Results:** The threshold value of CSASR for ultrasound diagnosis of NA was 1.55. The average diseased segments per patient was 2.49 ± 1.97, with an average overall length of 10.03 ± 7.95 cm. Nerve stereoscopic reconstruction could be conducted for swelling, torsion, incomplete constriction, and complete constriction.

**Conclusion:** The ultrasound image reconstruction method proposed in this study can accurately determine the site, range, and type of neuropathies in patients with NA, and simultaneously provide complete and accurate data information and intuitive morphological information.

## Introduction

Neuralgic amyotrophy (NA), also known as Parsonage-Turner syndrome, is a disease of peripheral nerves with acute onset that is accompanied by severe pain and may lead to severe dysfunction (Komatsu et al., 2020; Ortiz Torres et al., 2020; Ijspeert et al., 2021). Diagnosis by high-resolution magnetic resonance imaging (MRI) (Hilgenfeld et al., 2017; Sneag et al., 2017; Crim and Ingalls, 2017) and high-frequency ultrasonography (Pan et al., 2014; van Rosmalen et al., 2018) is used to identify the presence of edema in the nerve and its innervated muscles, and the presence of nerve constriction. There are four types of imaging diagnosis: swelling, torsion, incomplete constriction and complete constriction (Arányi et al., 2015; ArÁnyi et al., 2017). Despite the fact that MRI can be utilized for three-dimensional (3D) reconstruction of nerve tissues and ultrasound can be used for panoramic imaging of nerve tissues at present, they have the following limitations: 1. The boundary between the 3D neural imaging and surrounding tissue is blurred (Kim et al., 2007; Chaves et al., 2018; van Rosmalen et al., 2021). 2. Lack of objective data to determine whether a site is diseased, or accurately determine the range of a disease (Lieba-Samal et al., 2016; Bao et al., 2017; van Rosmalen et al., 2018). 3. The images cannot directly represent the degree of morphological variation and lesion distribution of the diseased nerve based on the individual differences (De Smet et al., 2013; Gstoettner et al., 2020a; Koh, 2021).

Given the shortcomings of the current NA imaging diagnostic methods, we propose a stereoscopic nerve reconstruction technique based on multiplanar bilateral contrast ultrasound images. The goal is to determine the range of the disease by setting ultrasound parameters and to provide clinicians with intuitive information on neuromorphological changes using a stereoscopic reconstruction model.

## Materials and methods

### Study design

In order to determine by ultrasound whether a particular part of a nerve is present with NA in a patient, in addition to identifying whether there is torsion and constriction, a criterion needs to be set to determine whether the swelling of the nerve is severe enough to cause symptoms. First, the ratio of the cross-section area (CSA) of the affected nerve measured by ultrasound to the CSA of the symmetric position on the normal side was defined as cross-section area swelling ratio (CSASR), which was used as an indicator to reflect the degree of nerve swelling. Secondly, the threshold of CSASR was calculated statistically through case-control studies of NA patients and normal controls, which was used as the basis for judging whether swollen nerves caused symptoms. Third, the scope of the disease was judged according to whether the CSASR of the diseased nerve was greater than or equal to the threshold and whether there was torsion and constriction. Finally, the neural stereoscopic model was reconstructed according to the CSA values of the affected and healthy nerves. At the same time, CSASR was used to reconstruct a stereoscopic model that could reflect the type, degree and extent of the lesions.

### Statistical analysis

We applied MedCalc 18.2.1 (MedCalc Software bvba, Ostend, Belgium) statistical software to draw the receiver operating characteristic (ROC) curves of CSASR data in case group and control group, and completed statistical calculation of CSASR critical value. *p* < 0.05 was considered statistically significant.

### Patient and control groups

We enrolled a total of 44 consecutive patients with NA who were diagnosed and treated at the Department of Hand Surgery of our hospital from July 2016 to June 2022. The patients’ first visit to the outpatient of our department took place during July 2016 to June 2020. Due to the COVID-19 pandemic, detailed ultrasound examination and stereoscopic reconstruction were not performed after June 2020. The numeric rating scale (NRS) was used to assess the level pain of patients on the patients’ initial visit to the outpatient of our department (Karcioglu et al., 2018). The total duration of pain before natural resolution was recorded for patients whose pain had already subsided. The disease course from onset to visit the outpatient of our department was recorded. The inclusion and exclusion criteria followed the NA diagnosis and exclusion criteria proposed by van Alfen in 2015 (van Alfen et al., 2015). On this basis, we set the patient’s first visit to the outpatient of our department as the inclusion criteria, and took the time from the onset of symptoms to the first visit to the outpatient of our department within 8 weeks as the inclusion criteria. At the same time, outpatient review and follow-up were set as exclusion criteria. No pain (Collins and Hadden, 2017) and normal muscle strength were set as exclusion criteria too. Seven patients had concurrent hypertension, 2 patients had lacunar infarction, and 6 patients had osteoporosis. All patients had no other concurrent diseases. We enrolled 30 healthy people without sensorimotor dysfunction as the control group. The control group underwent ultrasound examination in July 2018. The general information of patients and control group is shown in Table 1.

**TABLE 1:** General information.

-	Patients	Control group
Number of cases (n)	44	30
Male/Female (n)	30/14	17/13
Age (y) (mean ± SD)	50.3 ± 14.8	45.4 ± 15.1
Height (cm) (mean ± SD)	168.7 ± 5.8	166.7 ± 8.5
Weight (kg) (mean ± SD)	64.8 ± 8.3	67.0 ± 10.1
Left/Right (n)	22/23^b^	30/30
Mean NRS pain score (mean ± SD)	7.4 ± 0.7	0
Duration of pain (weeks) (mean ± SD)	2.9 ± 1.4^c^	0
Disease course^a^(weeks) (mean ± SD)	3.8 ± 2.1	0

^a^ The disease course varied from 1 to 8 weeks.

^b^ One of the cases involved both sides.

^c^ 27 patients who visited the hospital after the pain disappeared were counted the total duration of pain without intervention.

Ultrasonography and electrophysiology were performed on all patients within 3 days of visit. The ultrasonographic morphological characteristics of each patient with NA were classified. The Haishen NDI-092 electrophysiological/evoked potential meter (Haishen Medical Electronic Instrument Co., Ltd., Shanghai, China) was utilized for the neurophysiological examination of patients. The temperature of the distal limb was controlled at approximately 32°C. The nerves involved were found in the electrophysiological examination are shown in Table 2. Nerve conduction abnormalities found in the electrophysiological examination are shown in Table 3.

**TABLE 2:** Involved nerves found in the electrophysiological examination.

Involved nerves found in the electrophysiological examination	All cases	Number of cases with single-nerve involvement
Plexus of branch nerves	15	—
Long thoracic nerve	1^a^	0
Suprascapular nerve	7	0
Thoracodorsal Nerve	5	0
Medial anterior thoracic nerve	4	0
Axillary nerve	8	0
Musculocutaneous nerve	7	0
Radial nerve	31	15^b^
Median nerve	16	9^c^
Ulnar nerve	10	0
Medial antebrachial cutaneous nerve	3	0

^a^ Only 1 patient with a winged scapula had the long thoracic nerve tested in the electrophysiological examination.

^b^ Ultrasonography confirmed that 5 patients had only radial nerve trunk involvement, 2 had only the posterior interosseous nerve (PIN) involvement, and 8 had both radial nerve trunk and the PIN, involvement in these 15 patients.

^c^ Ultrasonography confirmed that 5 patients had only the anterior interosseous nerve (AIN) involvement, 4 had both median nerve trunk and the AIN, involvement, and 0 had only median nerve trunk involvement in these 9 patients.

**TABLE 3:** Nerve conduction abnormalities found in the electrophysiological examination.

	Total number (n) and proportion (%) of patients	Motor nerve amplitude abnormalities (n) (%)	Motor nerve conduction velocity abnormalities (n) (%)	Sensory nerve amplitude abnormalities (n) (%)	Sensory nerve conduction velocity abnormalities (n) (%)
All patients (the abnormalities in nerve entrapment sites were not counted)	44, 100%	44, 100%	7, 15.9%	5, 11.4%	5, 11.4%
Patients with concurrent nerve entrapment (only the abnormalities in nerve entrapment sites were counted)	4^a^, 100%	2, 50.0%	3, 75.0%	4, 100%	4, 100%
Patients with secondary nerve entrapment (only the abnormalities in nerve entrapment sites were counted)	4^b^, 100%	1, 25.0%	2, 50.0%	3, 75.0%	4, 100%

^a^ 4 patients had nerve entrapment symptoms at or before the onset of pain. 3 of them had concurrent carpal tunnel syndrome (CTS), and 1 had cubital tunnel syndromec (CuTS) according to the electrophysiological examination.

^b^ 4 patients had nerve entrapment symptoms after the onset of pain. 2 of them had secondary CTS, and 2 had secondary CuTS., according to the electrophysiological examination.

The outpatient physicians, ultrasound examiners, and electrophysiological examiners were double-blinded and did not disclose patient information or examination results to each other. All the ultrasonic examinations were performed by an ultrasound department director in our hospital with more than 10 years of experience in neuroultrasound.

### Ultrasonographic examination methods

Examinations were performed utilizing the Siemens ACUSON S2000 ultrasound system (Siemens AG, Munich, Germany) Philips iU22, and EPIQ7C ultrasound device (Philips Electronics, Amsterdam, Netherlands) using probes with frequencies of 12–18 MHz. The nerves on the affected side of the patients were scanned using axial ultrasound, and the normal side was used as a control to preliminarily determine the location and range of the aberrant nerve morphology on the affected side. CSA was measured at multiple symmetrical locations along the entire length covering all the disease sites of both affected and contralateral nerves. The CSA measurement plane is perpendicular to the nerve axis, which is the minimum measurement area of the same axial point. To better reflect the details of the morphological changes, the distance between the measurement sites with mild morphological changes in the nerves was set at 3.0–5.0 cm, and the distance between the measurement sites with sharp changes in abnormal morphology was set at 0.3–0.5 cm. CSA was randomly measured at eight symmetrical positions of the brachial plexus nerves for each and every person in the control group.

### Ultrasound-based diagnostic method

#### Diagnostic threshold value of cross-section area swelling ratio

CSASR was calculated as follows: CSASR = CSA of the nerve on the affected side/CSA of the nerve on the normal side at the same level. The CSASR reflects the degree of swelling of the diseased nerve in comparison to the nerves on the normal side.

The maximum cross-sectional area swelling ratio values of all 44 patients and 30 normal controls were calculated, moreover, the ROC curve was plotted using MedCalc 18.2.1 statistical software (MedCalc Software bvba, Ostend, Belgium) to evaluate the diagnostic threshold value, sensitivity, specificity, area under the ROC curve (AUC), and *p*-value.

#### Defining the range of neuropathy

Scanned locations with a CSASR ≥ the diagnostic threshold value were considered the diseased locations. The nerve segment with consecutive diseased sites was considered as the diseased nerve segment. The range of the disease is defined as the sum of the diseased nerve segments along with the site of the constriction rings found on the axial scan.

#### Stereoscopic model reconstruction

Reconstruction of the nerves on the affected and normal sides using a stereoscopic model:

The left and right sides of each ultrasound cross-section images were cropped, so that the distance between the midpoint of the nerve cross-section and the two lateral boundaries of the image were equal, and the sizes of all cropped ultrasound images were the same. Unigraphics NX 1875 software (Siemens PLM Software, Ostend, Belgium)was used to arrange the cropped ultrasound images parallel to each other and to reconstruct the model of the nerve on the affected side and the normal side. The images were positioned according to the distance along the long axis of the limb between each scanning site and the anatomical landmark on the patient’s body. The curvature of the nerve model was adjusted to match the curvature of the spliced image of the axial nerve ultrasonography in order to make the visual effect consistent between the two.

CSASR model reconstruction:

The circular CSA of the model was calculated using the CSASR value for each ultrasound scanning site. Combining this with the distance between each ultrasound scanning site and the anatomical landmark on the patient’s body along the long axis of the limb, we used Unigraphics NX 1875 (Siemens PLM Software, Ostend, Belgium) to reconstruct an unequal thickness tubular CSASR model. Geomagic Wrap 2017 software (3D Systems, Inc., Rock Hill, SC, United States) was used to automatically identify whether the CSA of the CSASR model exceeded the threshold value. The parts of the model with their CSA less than the threshold value were colored dark blue, while the parts of the model with their CSA greater than or equal to the threshold value were colored with a gradient ranging from green to red based on the overage. The curvature was modified to be consistent with the curvature of the spliced image of the nerve ultrasonography. By keeping the length of the model unchanged, the overall thickness of the model was altered proportionally so that its visual effect was similar to that of the nerve stereoscopic reconstruction model.

#### Ultrasound classification

The ultrasonographic morphological characteristics of each patient with NA were classified, and the morphological characteristics, number of cases, proportion, and decreased muscle strength (Medical Research Council Muscle Scale) of each type were summarized.

This study was approved by the Ethics Committee of Ningbo No.6 Hospital (No. L2016034).

## Results

### CSASR diagnostic threshold value

The ROC curve was used to calculate the CSASR diagnostic threshold value for patients and the results are shown in Table 4.

**TABLE 4:** Diagnostic cut-off value.

Mean of the patients’ maximum CSASR values (mean ± SD)	Mean of the control group’s maximum CSASR values (mean ± SD)	CSASR diagnostic threshold value	Sensitivity (%)	Specificity (%)	AUC	*p*-value
2.73 ± 1.41	1.37 ± 0.16	1.55	95.4	86.67	0.973	<0.001

### Neuropathy range

A total of 39 patients were included in the statistical analysis. Due to the interference of the clavicle, the length of the diseased nerve was not measured in five patients with ultrasound-detected swelling of the brachial plexus roots alone. A total of 45 nerves with significant symptoms were tested by ultrasonography in the 39 patients. Among them, 2 nerves were scanned in 3 patients, and 4 nerves were scanned in 1 patient. The 45 nerves included 26 radial nerves, 15 median nerves, 3 ulnar nerves, and 1 musculocutaneous nerve.

The average number of diseased nerve segments among the 39 patients with NA was 2.49 ± 1.97, and the average total length of diseased nerve segments was 10.03 ± 7.95 cm. In the 45 diseased nerves, the average number of diseased nerve segments was 2.16 ± 1.31, and the average total length of diseased nerve segments of each nerve was 8.69 ± 6.87 cm. In the 7 patients with a constriction ring, only the sites with a cross-sectional area swelling ratio ≥1.55 were counted. Seven patients had a total of 20 constriction rings, so the average number of constriction rings per patient was 2.86 ± 1.57.

### Ultrasound classification

The ultrasonographic findings and decreased muscle strength of the 44 patients are summarized into four types and displayed in Table 5.

**TABLE 5:** Ultrasound classification and the patient’s muscle strength decline.

Type	Ultrasound findings	Number of cases and proportion (%)	Number of patients when the minimum muscle strength reaches different levels				
			M0	M1	M2	M3	M4
Type I, swelling	• Longitudinal scan showed that the nerve was swelling, the epineurium was flat, and the higher-echogenicity line of the nerve bundles was in line with the direction of the epineurium	32, 72.7%	2	1	7	15	7
	• The cross-sectional scan showed that the CSA of the nerve on the affected side was larger than that of the normal side, and there was no rotation of the nerve bundle						
Type II, torsion	• Longitudinal scan showed that the nerve swelling, the epineurium was uneven with wave-like appearance, and the higher echogenic line of the nerve bundle membrane was inconsistent with the direction of the epineurium	5, 11.4% (1 case of posterior interosseous nerve, 4 cases of both radial nerve trunk and the PIN)	3	0	1	1	0
	• In slow dynamic cross-sectional scans, the swollen nerve bundle may be seen to rotate clockwise or counterclockwise						
Type III, incomplete constriction	• Longitudinal scan showed that there was one or more constriction rings, and the hypoechoic nerve bundle that was narrower than that of the normal side passed through these constriction rings	3, 6.8% (1 case of musculocutaneous nerve, 2 cases of both radial nerve trunk and the PIN)	2	0	1	0	0
	• Cross-section scan showed that the hypoechoic nerve bundle at the constriction ring was significantly thinner than that on the normal side, and the nerve at the proximal and distal end of the constriction ring was thickened						
Type IV, complete constriction	• Longitudinal scan showed that there was one or more constriction rings, and the hypoechoic nerve bundle at the constriction ring was broken	4, 9.1% (2 cases of the AIN, 2 cases of both radial nerve trunk and the PIN)	2	2	0	0	0
	• Cross-section scan showed that the hypoechoic nerve bundle disappeared completely at the constriction ring, and the nerve at the proximal and distal end of the constriction ring was thickened						

### Nerve stereoscopic reconstruction of typical cases

*A typical type I case: Nerve swelling*

**FIGURE 1:**
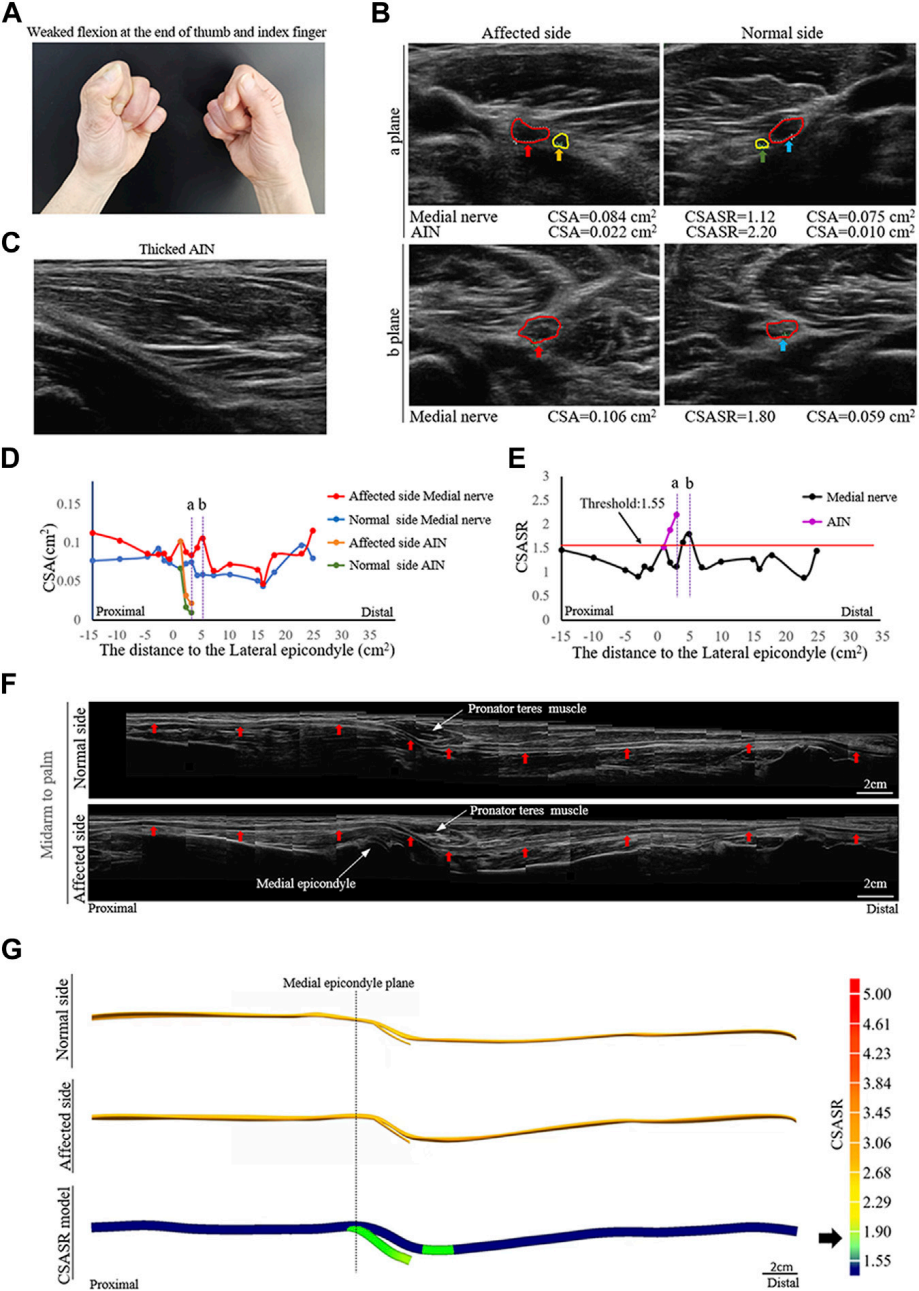
A typical NA case of nerve swelling. **(A)** Weak flexion of the distal phalanx of the right thumb and index finger. **(B)** Comparison of the cross-section area (CSA) of the medial nerves and the anterior interosseous nerves (AINs) on the normal and affected sides at sites (a, b) in panels **(D,E)**. The circle in red indicates the median nerve. The circle in yellow indicates the AIN. **(C)** In the axial ultrasound image, the starting segment of the affected AIN is thickened. **(D)** A comparison of the CSA of each ultrasound scan site on the normal and affected sides. **(E)** The cross-section area swelling ratio (CSASR) of each ultrasound scanning site. **(F)** A comparison of the spliced images of the axial ultrasound scans between the normal side and the affected side. The red arrows indicate the median nerve. **(G)** The ultrasound stereoscopic reconstruction model of the normal and affected nerve as well as the CSASR reconstruction model. The green areas represent the range of disease and degree of nerve swelling in the CSASR reconstruction model.

A patient diagnosed with right upper extremity onset and median nerve symptoms as the main manifestation (Figure 1).

*A typical type II case: Torsion nerve*

**FIGURE 2:**
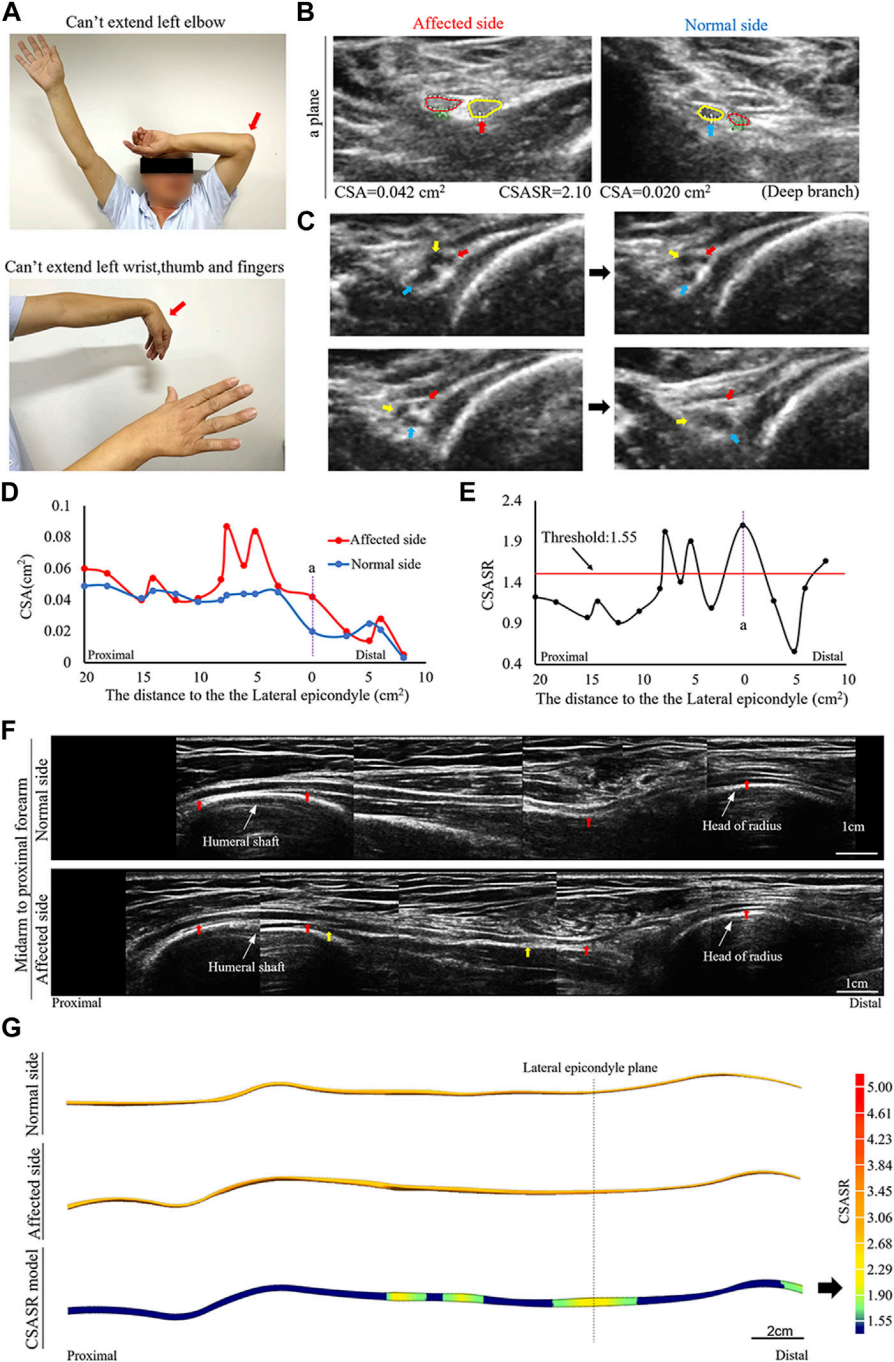
A typical NA case of torsion nerve. **(A)** Inability to perform elbow extension, wrist extension, finger extension, as well as thumb extension. **(B)** The CSA of the radial nerves on the affected and normal sides are compared at site (a) in panels **(D,E)**. The red circle indicates the superficial branch of the radial nerve. The circle in yellow shows the posterior interosseous nerve (PIN). **(C)** Continuous cross-section scanning of 6–7 cm proximal to the lateral epicondyle revealed three swollen nerve bundles in the radial nerve trunk that were twisted counterclockwise. The direction of the arrow passes through the midpoint of the nerve bundle and the midpoint of the nerve trunk. **(D)** Comparison of the CSA on the affected side with that on the normal side in each ultrasound scanning site. **(E)** The CSASR of each ultrasound scanning site. **(F)** A comparison of the spliced images of the axial ultrasound images on the affected side and the normal side. The red arrow indicates the radial nerve leading to the PIN. The epineurium of the radial nerve between the yellow arrows is uneven and has wave-like changes, and the direction of nerve bundle membrane is inconsistent with the direction of the epineurium. **(G)** The ultrasound stereoscopic reconstruction model for nerves and the CSASR reconstruction model. The green to yellow parts show the range of disease and the degree of nerve swelling in the CSASR reconstruction model.

A patient diagnosed with left sided onset and had radial nerve symptoms as the main manifestation (Figure 2).

*A typical type III case: Incomplete constriction nerve*

**FIGURE 3:**
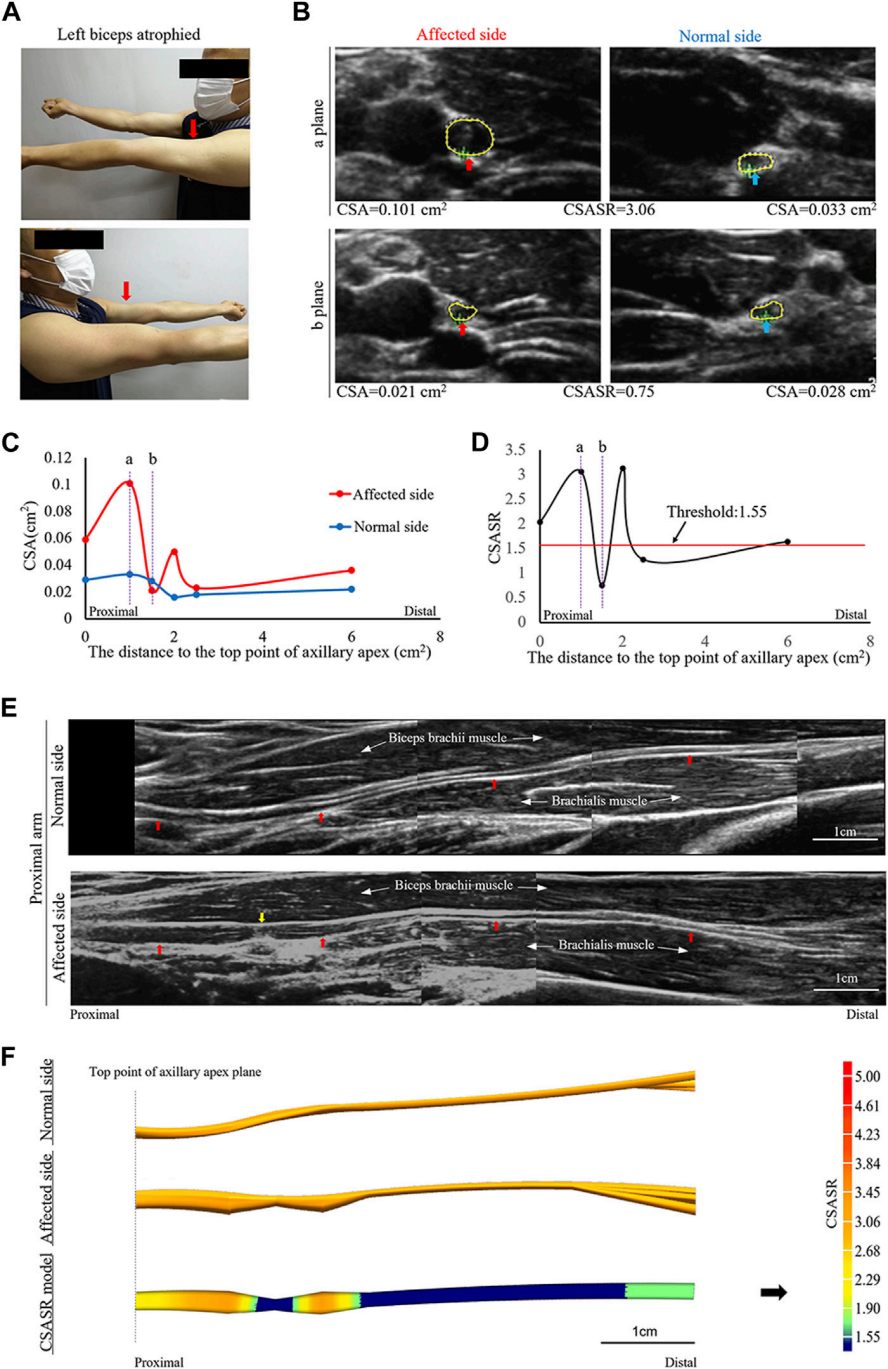
A typical case of incomplete nerve constriction in NA. **(A)** Left biceps atrophy. **(B)** The CSA of the musculocutaneous nerves on the affected and normal sides are compared at sites (a, b) on panels **(C,D)**. **(C)** Comparison of the CSA of the nerves on the affected and normal sides at each ultrasound scan location. The CSA value of the most distal location is the sum of the CSA values of all the branches. **(D)** CSASR for each ultrasound scan location. **(E)** Comparison of the spliced images of the axial ultrasound scans between the affected side and the normal side. The red arrow indicates the musculocutaneous nerve, and the yellow arrow indicates the constriction ring. **(F)** The ultrasound stereoscopic reconstruction and CSASR reconstruction models of the nerve. The green to orange parts represent the range of disease and the degree of nerve swelling in the CSASR reconstruction model.

A patient was affected on the left side and had musculocutaneous nerve symptoms as the main manifestations (Figure 3).

*A typical type IV case: Complete constriction nerve*

**FIGURE 4:**
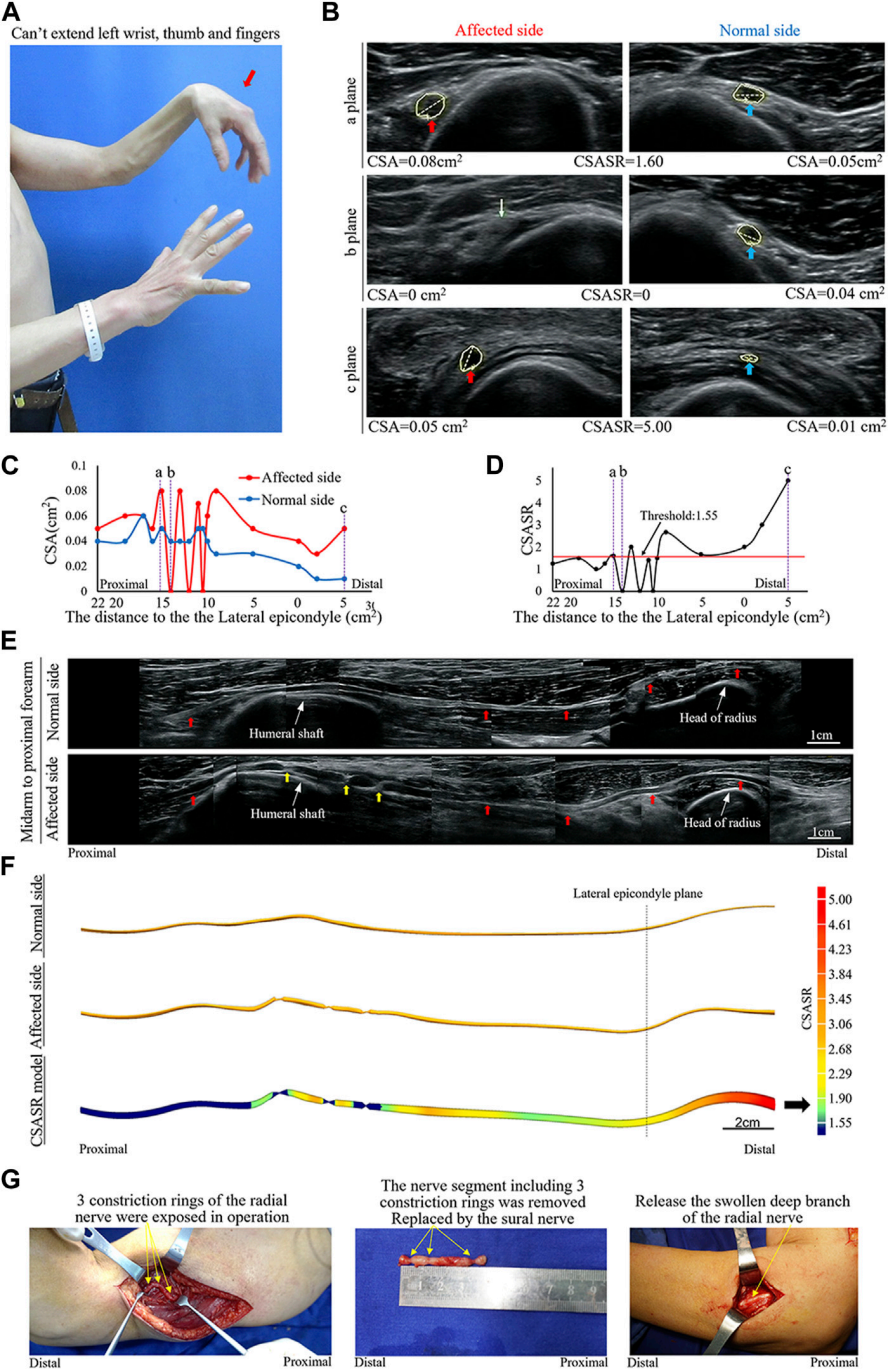
A typical NA case of complete constriction nerve. **(A)** Inability of left wrist extension, finger extension, and thumb extension. **(B)** The CSA of the radial nerves is compared between the affected and normal sides at sites a, b, and c in panels **(C,D)**. The yellow arrow indicates the disappearance of the nerve bundle at the constriction ring. **(C)** Comparison of the CSA of each ultrasound scan site on the affected and normal sides. **(D)** CSASR for each and every ultrasound scan site. **(E)** Comparison of the spliced images of the axial ultrasound scans between the affected and normal sides. The red arrow indicates the radial nerve to the PIN, whereas the yellow arrow indicates the three constriction rings of the radial nerve. **(F)** The ultrasound stereoscopic reconstruction model and the CSASR reconstruction model of the nerve. The green to red parts show the range of disease and the degree of nerve swelling in the CSASR reconstruction model **(G)** Three constriction rings were detected in the radial nerve of the upper arm during the operation and the PIN was swollen.

A patient was affected on the left side and had radial nerve symptoms as the main manifestations (Figure 4).

## Discussion

We reconstructed a stereoscopic model with a localization function that reflected the degree of neuromorphic changes by integrating the two-dimensional ultrasound scanning plane graphics and data from multiple sites. According to the diagnostic threshold value of CSASR obtained in this case-control study, it is possible to visually reflect the distribution of lesions, determine the range of disease, and describe the severity of lesions. This makes up for the current deficiencies in neuroimaging diagnosis and provides a novel approach for data visualization of imaging-based diagnosis using existing instrument technologies.

In this study, we used the multilocational bilateral contrast method for the ultrasound examination of the patients. Since the symmetry of the CSA of human bilateral nerves has been confirmed (Thoirs et al., 2008; Ulaşli et al., 2013; Lämmer et al., 2015), the individual differences of CSA appear symmetrically on both the affected and normal sides. Assigning the CSASR as a diagnostic index could offset the individual differences in the CSA between the affected and normal sides (Bedewi et al., 2017; Li et al., 2019). Among patients with NA, 97% have bilateral asymmetry (Seror, 2017), hence, the proposed ultrasound-based diagnostic method is applicable for the vast majority of patients with NA.

In this study, the statistically determined CSASR threshold value was established as a unified standard for ultrasound diagnosis of NA, and defined the range of the nerve disease outside the constriction ring based on nerve segments with a CSASR greater than or equal to the threshold. All patients included in this study had a disease course of less than or equal to 8 weeks, and the diagnostic criteria of patients with a disease course of more than 8 weeks may be biased from the results of this study. In this study, the standard deviations of the average number of diseased nerve segments in the upper limb of each patient and the average total length of the diseased nerve segments of each patient were very large, which suggest that the location and length range of NA are random and not regular, which is consistent with the characteristics of NA described in literature—one or multiple mononeuropathies with multifocal distribution (Collins and Hadden, 2017; Sneag et al., 2018).

We used ultrasound instruments to accurately trace the boundary of the nerve cross-section and reconstruct the stereoscopic model, which accounted for the defects of fuzzy boundary in existing neural 3D image and difficulties in measuring morphological data accurately. Due to the transverse displacement of the ultrasound probe with the anatomical position of the nerve during the examination, and the pressure placed by the examiner on the probe, the nerve could be displaced. The reconstruction of the model in this study was not a three-dimensional reconstruction of the actual shape of the nerve, but a stereoscopic model of the rough projection of the nerve on the sagittal site. The positioning of the cross-section of the model along the direction of the long axis of the limb is consistent with the cross-section of the actual nerve as determined by ultrasound. Although the CSASR reconstruction model is not a direct simulation of nerve morphology, it is a stereoscopic representation of the degree of variation in the diseased nerve morphology relative to normal morphology.

NA is a clinical diagnosis. Currently, general practioners give patients analgesic (Van Eijk et al., 2016) and oral corticosteroids (Van Alfen and van Engelen, 2006) out of hospital in the acute phase and may last up to 3 months (Ochi et al., 2013; Wu et al., 2014) from the onset of symptoms. If spontaneous recovery is not inadequate, the patient go to the hospital for ultrasound or MRI to determine whether the nerve constriction is present. Patients with neuroconstriction should consider surgery. Surgery includes nerve release, nerve suture and nerve graft (Pan et al., 2014; Wang et al., 2019). Clemens Gstoettner (Gstoettner et al., 2020b) counted 143 patients with neuroconstriction, including 12 different studies. 122 (85.3%) patients recovered muscle strength to M4 or above. Most patients underwent surgery 3–6 months after onset, and 9 patients (6.3%) had surgery 1 year or more after onset, of which only 6 patients (66.7%) recovered well. This indicates that the time of surgical intervention has an effect on the therapeutic effect (ArÁnyi et al., 2017). Early detection of neuroconstriction through imaging and surgical intervention may result in a better prognosis. In this study, we statistically found that patients with neuroconstriction had more severe muscle loss than those without. The muscle strength of all patients with constriction decreased to M0-M2 at the time of presentation within 2 months of onset. Muscle strength decreased to M0-M2 in 75.0% of patients with torsion. Only 31.3% of patients of swelling type had muscle strength reduced to M0-M2. Therefore, when the muscle strength of NA patients is found to decrease to M0-M2, immediate imaging exploration is necessary. In addition, neuroconstriction undergoes a dynamic process of formation, and multiple imaging exploration at different time points may provide useful information on the course of the disease (Gstoettner et al., 2020b).

There are many factors affecting the prognosis of NA patients that can be detected by ultrasonography. The factors that can be determined to be associated with the prognosis of NA patients are the severity of neuroconstriction (Wu et al., 2014; Sunagawa et al., 2017; Wang et al., 2019) and the management of concurrent or secondary nerve entrapment. Due to the multifocal distribution of NA, it is necessary to explore the full length of the diseased nerve during ultrasound examination, and the omission of serious lesion sites may affect the prognosis of patients. Nerve swelling, as the most common ultrasound manifestation of NA, has been described digitally by CSASR calculation in this study. Does the severity of swelling affect patient prognosis (Gstoettner et al., 2020b)? Can ultrasound-guided precise corticosteroid injection be used to reduce the side effects of systemic hormone therapy? or to further increase the efficacy on the basis of systemic hormone therapy (Yang et al., 2021; Wright et al., 2023)? Can increased introneural pressure due to severe swelling be used as an indicator of surgical intervention? These questions need to be confirmed by further clinical studies. The ultrasonography and reconstruction of the stereomodel in this study can reflect all the prognostic factors mentioned above. However, conventional ultrasound and MRI reports provide insufficient data information and lack the general boundary between diseased nerve and normal nerve, which will not help with the precision treatment that may be implemented in the future.

The following are the shortcomings of this study. This was a retrospective study. The patients who were examined by the sonographer were all patients from the hand surgery clinic. The incidence of radial nerve and median nerve neuropathy was the highest, which differs from the results of previous studies that suggest the incidence of suprascapular and long thoracic nerve neuropathy to be the highest (Ferrante and Wilbourn, 2017; Sneag et al., 2018) The patients involved in this study were only 44 cases in nearly 4 years from July 2016 to June 2020, especially only 8 cases before January 2018 in our specialized orthopedic hospital with the average annual outpatient volume closed to 70,000. There is a big gap with the annual incidence approximately 1/1,000 reported by van Alfen (van Alfen et al., 2015), which indicating that doctors’ awareness of the disease was not widespread at this stage. The enrolled patients may not be representative of the entire NA patient population, hence the study needs to be further verified by prospective, multicenter studies. Ultrasound examination was only performed on diseased nerves with obvious clinical manifestations, and several nerves with subclinical abnormalities were not included. There was no relevant disease control in the control group, so there was a certain deviation in the specificity of the diagnostic threshold value of CSASR. In addition, different ultrasonic instruments with their own individual reference values will produce deviation to the measurement results. Experience of the person performing the ultrasound measurements will also affect the accuracy of the measurements. So it’s necessory to reflect on the technique with multiple assessors over multiple centers, when clinically implemented in future. Finally, this method cannot be applied to diagnose the few cases in which the bilateral nerves develop disease simultaneously at the same level.

## Conclusion

The ultrasound examination and model reconstruction methods proposed in this study can provide clinicians with full range, intuitive and digitized lesion information. On this basis patients will receive more accurate and effective treatment. In the future, patients will benefit more from gradually digitized imaging diagnosis.

## Data Availability

The original contributions presented in the study are included in the article/Supplementary Material, further inquiries can be directed to the corresponding author.
